# Identification of risk areas for avian influenza outbreaks in domestic poultry in Mali using the GIS-MCDA approach

**DOI:** 10.1017/S0950268824001390

**Published:** 2024-12-02

**Authors:** Idrissa Nonmon Sanogo, Maxime Fusade-Boyer, Sophie Molia, Ousmane A. Koita, Christelle Camus, Mariette F. Ducatez

**Affiliations:** 1 Interactions Hôtes-Agents Pathogènes (IHAP), UMR 1225, Université de Toulouse, ENVT, INRAE, 31076 Toulouse, France; 2 Faculté d’Agronomie et de Médecine Animale (FAMA), Université de Ségou, Ségou BP 24, Mali; 3 CIRAD, UMR ASTRE, F-34398 Montpellier, France; 4 ASTRE, Université de Montpellier, CIRAD, INRAE, Montpellier, France; 5 Laboratoire de Biologie Moléculaire Appliquée (LMBA), Université des Sciences, des Techniques et des Technologies de Bamako, Bamako, Mali

**Keywords:** influenza a virus, poultry, risk mapping, multi-criteria decision analysis, geographic information system, Mali

## Abstract

Mali is a country where little information is known about the circulation of avian influenza viruses (AIVs) in poultry. Implementing risk-based surveillance strategies would allow early detection and rapid control of AIVs outbreaks in the country. In this study, we implemented a multi-criteria decision analysis (MCDA) method coupled with geographic information systems (GIS) to identify risk areas for AIVs occurrence in domestic poultry in Mali. Five risk factors associated with AIVs occurrence were identified from the literature, and their relative weights were determined using the analytic hierarchy process (AHP). Spatial data were collected for each risk factor and processed to produce risk maps for AIVs outbreaks using a weighted linear combination (WLC). We identified the southeast regions (Bamako and Sikasso) and the central region (Mopti) as areas with the highest risk of AIVs occurrence. Conversely, northern regions were considered low-risk areas. The risk areas agree with the location of HPAI outbreaks in Mali. This study provides the first risk map using the GIS-MCDA approach to identify risk areas for AIVs occurrence in Mali. It should provide a basis for designing risk-based and more cost-effective surveillance strategies for the early detection of avian influenza outbreaks in Mali.

## Introduction

Wild waterfowl are considered the natural reservoir for avian influenza viruses (AIVs), which can be responsible for highly contagious and severe illnesses in birds. These viruses are classified as either high pathogenicity (HPAI) or low pathogenicity (LPAI) viruses depending on their virulence in poultry [1]. LPAI subtypes, although less virulent in birds, may represent a gene pool for the emergence of new genotypes of HPAI viruses by reassortment, a powerful evolutionary mechanism used by influenza viruses [2–4]. Because of their important genetic diversity and ability to evolve rapidly, AIVs represent a major concern for human and animal health worldwide. Indeed, AIVs may cross the species barriers and infect new hosts, including humans [5, 6]. HPAI viruses are responsible for high morbidity and mortality, especially in the poultry industry, causing huge economic losses that can compromise food security in the South [7]. Since its first detection in Nigeria in 2006, HPAI viruses have been responsible for large outbreaks in several African countries over the past 15 years [8].

In West Africa, Mali first notified HPAI outbreaks to the World Organization of Animal Health in March 2021, despite evidence of AIVs circulation being reported since 2007 [9]. These outbreaks mainly affected the commercial poultry sector and caused huge economic losses to farmers. Although there is limited information on the epidemiology of AIVs in Mali, the country was considered to be at high risk for infection with HPAIV because it is surrounded by countries that have experienced HPAI outbreaks, in a context where illegal trade and cross-border movements of poultry are common [10]. Furthermore, the Inner Delta of the Niger River (the second largest continental wetland in Africa) is located in Mali, which provides suitable breeding and resting sites for millions of migratory birds potentially carrying AIVs [11, 12]. In Mali, more than 90% of the domestic poultry live in traditional farming systems where biosecurity measures are not observed [13], and the detection of animal disease outbreaks such as HPAI relies mainly on passive surveillance. Active surveillance is very limited and depends on external funding [14]. In such a situation, where there is a lack of reliable information on the circulation of animal diseases, risk-based surveillance activities should facilitate early detection and a quicker outbreak response [15]. Methods such as GIS-based multicriteria decision analysis (GIS-MCDA) could contribute to the implementation of risk-based surveillance strategies in Mali by helping identify areas where surveillance and control activities should be targeted and thus contribute to the efficient use of the limited resources available for surveillance activities. GIS-MCDA allows the combination of data on risk factors linked to a particular disease and spatial data to produce a risk or suitability map for disease occurrence [16]. In addition, this method may be appropriate in data-scarce conditions, serving as an alternative to data-driven methods for risk mapping [17]. The GIS-MCDA approach has been applied to identify suitable areas for the occurrence of influenza A and D viruses in Africa, Asia, and Latin America [18–20].

In the present study, we aimed to implement a GIS-based MCDA method to identify areas at risk for AIVs occurrence in domestic poultry in Mali. The output of this study could serve as a basis for designing risk-based and more cost-effective surveillance strategies for AIVs.

## Materials and methods

### Study framework

In this study, we performed a GIS-based MCDA approach for risk mapping of avian influenza viruses (AIVs) occurrence in Mali. The GIS-MCDA method was performed in five main steps: (1) identification of risk factors related to avian influenza outbreaks; (2) determination of the relative weight of each risk factor; (3) collection of spatial data on risk factors, standardization, and geoprocessing; (4) generation of final AI risk maps by the weighted linear combination of risk factor maps and zonal statistics; and (5) uncertainty, sensitivity analysis, and risk map validation.

### Avian influenza risk factors

We carried out a systematic literature review as explained elsewhere [21]. Briefly, searches were performed in two scientific databases (PubMed and Cab abstract) with the keywords ‘Avian influenza’ AND ‘risk factors’ AND ‘Africa’ to identify risk factors linked to the occurrence of AIVs in poultry in Africa. Two selection criteria were considered when selecting the risk factors: The first was their relevance to the epidemiology of avian influenza in the region, that is, if they had been repeatedly reported to be significantly associated with the occurrence of AIVs, and the second was the possibility of obtaining spatial data on the given risk factor. From the literature review, we identified five main risk factors (Table 1) that are potentially associated with avian influenza outbreaks in West Africa.

**Table 1.** Risk factors associated with avian influenza outbreaks

Risk factors	Hypothesis/Explanation	References
Poultry density	AIVs are transmitted through direct contact between infected and susceptible birds, therefore high poultry density is expected to increase the risk of Avian Influenza outbreaks.	[26, 27]
Proximity to poultry markets	Live bird markets represent a place where domestic poultry from various origins, breeds, and ages are brought together for purchase and their proximity is therefore considered a risk factor for Avian influenza outbreaks.	[28, 37]
Proximity to commercial poultry farms	Proximity to commercial poultry farms may involve not only the mixing with other birds but also the potential exchange of equipment such as egg crates between farmers. Therefore, proximity to infected commercial poultry farms is expected to increase the risk of Avian Influenza outbreaks.	[29]
Proximity to roads	Proximity to major roads was associated with AI outbreaks. The movement of domestic poultry and their products along major road networks could contribute to the spread of AIVs.	[38, 39]
Proximity to waters areas	Proximity to water bodies is associated with an increased risk of avian influenza outbreaks by favouring contact between domestic poultry and wild birds, which are considered to be AIVs reservoirs.	[11, 12]

### Expert survey and risk factors weights

An electronic questionnaire (Supplementary Table S2) developed on Google Forms was submitted to 14 local and international experts (academics, researchers and members of non‐governmental, governmental and international organizations) who have published or are actively working on AIVs in the West African region. Experts were asked to fill in a pairwise comparison matrix, where each risk factor was compared with the others, according to its relative importance, on a five-point scale ranging from 1/5 (“much less important”5), through 1 (“equally important”) to 5 (“much more important”). The experts were also asked to select from the list of four mathematical functions (linear, quadratic, sigmoidal, and linear bidirectional) the type of relationship between each risk factor and the occurrence of AIVs and to determine the threshold values corresponding to ‘negligible,’ ‘very low,’ ‘low,’ ‘moderate,’ ‘high,’ ‘very high’ risk. We used the analytical hierarchy process (AHP) to assign a weight to each risk factor [22]. The consistency of each pairwise comparison was evaluated by calculating the consistency ratio (CR) using the following equation (1):
(1)

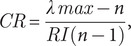

where *λmax* is the maximal eigenvalue of the pairwise comparison matrix, n is the number of factors, and RI is a random index [22]. CR < 0.10 indicates that the pairwise comparison matrix has an acceptable consistency and the derived weights can be used for further analyses. However, if CR ≥ 0.10, that indicates inconsistent judgement, then the pairwise comparison should be revised [22]. Thus, experts with inconsistent judgements were asked to re-examine their pairwise comparison matrix.

### Spatial data collection, standardization, and geoprocessing

Spatial data were collected for each risk factor from various sources (Table 2) and processed to produce standardized raster layers (Supplementary Figure S1–S5) with cell values ranging from 0 (negligible risk) to 5 (very high risk). Poultry density was extracted from spatial data layers from the Gridded Livestock of the World in the unit of number of birds/km^2^ [23]. Distance to poultry markets, commercial poultry farms, roads, and water bodies was calculated using their geographical centroid and transformed into a geographical layer in kilometres units with Euclidean distance, assigning greater risk to areas close to the centroids (Table 2). Finally, standardised risk factor maps were calculated using fuzzy linear and sigmoidal membership functions on a scale of 0–5 (unsuitable to perfectly suitable). All spatial data were processed and transformed using ArcGIS 10.2 (ESRI, Redlands, CA, USA). After the geoprocessing step, we obtained for each risk factor a standardized raster layer with a resolution of 1,000 × 1,000 m.

**Table 2.** Standardisation and reclassification of geographical layers

Risk factors	Data source	GIS processing	Scaling function
Poultry density	[23] https://www.geo-wiki.org/	Calculate poultry density (nb animal/km^2^) Standardisation (extent, pixel size) Reclassification with fuzzy linear membership function	Positive linear relationship **Reclassification units** > 5,000 poultry/km^2^: Very high risk (5) 500–5,000 poultry/km^2^: High risk (4) 200–500 poultry/km^2^: Moderate risk (3) 100–200 poultry/km^2^: low risk (2) 50–100 poultry/km^2^: Very low risk (1) 0–50 poultry/km^2^: Negligible risk (0)
Proximity to poultry markets	[28]	Euclidean distance (km) to the feature of interest (Poultry markets, commercial poultry farms, road, and water areas) Standardisation (extent, pixel size) Reclassification with fuzzy sigmoidal membership function at a scale of 0–5 (unsuitable to perfectly suitable).	Sigmodal, monotonically decreasing relationship with highest risk within 0–5 km from poultry market, commercial poultry farms, road, and water areas afterward AIV risk decreased with negligible risk after 10 km. **Reclassification units** < 2 km: Very high risk (5) 2–4 km: High risk (4) 4–6 km: Moderate risk (3) 6–8 km: low risk (2) 8–10 km: Very low risk (1) > 10 km: Negligible risk (0)
Proximity to commercial poultry farms	[10]		
Proximity to roads	[40] https://www.globio.info/global-patterns-of-current-and-future-road-infrastructure		
Proximity to water areas	DIVA - GIS https://www.diva-gis.org/datadown		

### Generation of the avian influenza risk maps

An avian influenza (AI) risk map was generated with the raster calculator tool of the ArcGIS 10.4.1 (ESRI, 2017) software by performing a weighted linear combination (WLC) of the standardized spatial layers of risk factors as shown in equation (2):
(2)

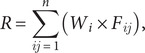

where *R* indicates the risk index estimate for each raster cell, *n* is the number of risk factors, *W_i_* is the weight for risk factor I, and *F_ij_* is the value of risk factor *i* for raster cell *j.*

The AI risk map was finally presented on a graduated blue-green-yellow-orange-red scale, ranging from 0 to 5. We have also calculated the average risk of AI outbreaks for each municipality by performing zonal statistics on the final AI outbreaks risk map using ArcGIS 10.4.1 (ESRI, 2017).

### Uncertainty, sensitivity analysis and risk map validation

A sensitivity analysis was conducted using a method previously described [24] to assess the robustness of the AI risk map to changes in the relative weights assigned by experts to each risk factor. Briefly, a total of 10 scenarios were constructed by increasing and decreasing the weights of each risk factor by a total of 25% of their initial value. The weights of the other risk factors were adjusted so that the sum of all the weights was equal to one. For each scenario, a new risk map was generated, and the average risk value was calculated for all pixels. We used non-parametric Spearman correlation coefficients (Rho) to compare the final AI risk map with the outputs obtained in each of the 10 scenarios. A change of <10% in the correlation coefficients indicated evidence of the robustness of the model to changes in the weights of risk factors [24]. In addition, to evaluate the contribution of each risk factor to the variability of the final risk estimate, a multiple regression model without intercept was fitted using the average risk index from the output maps of the 10 scenarios as the dependent variable and the risk factor weights as predictors [20]. The contribution of factor i to the variation in the risk index was the standardized coefficient estimate associated with i divided by the sum of the absolute value of the standardized coefficients of all the risk factors. Thus, higher coefficients indicate a greater influence of the risk factor on the final risk estimate. Moreover, an uncertainty map was produced by calculating the standard deviation of the different risk maps resulting from the changes in weights [20].

The sensitivity analyses were conducted using the statistical software RStudio version 2022.12.0 + 353 running on R version 4.2.2 (R Development Core Team, 2021) and QGIS 3.24 (QGIS Development Team, 2022). The MCDA model was validated by overlaying the reported outbreak locations from the EMPRES-i database on the generated AI risk map. Complete risk map validation was not possible due to the lack of field data regarding the occurrence of AIV in Mali, nevertheless, the risk map was compared with the location of the few HPAI outbreaks already reported in Mali.

## Results

### Risk factors weights

The relative weights of the risk factors associated with AI outbreaks in Mali are presented in Table 3.

**Table 3.** Weights attributed by the experts

Risk factors	Relative weights (%)
Poultry density	31.33
Proximity to poultry markets	23.07
Proximity to commercial poultry farms	25.62
Proximity to water areas	11.33
Proximity to roads	8.65

The result of the AHP application showed that experts (9 had fully replied to the questionnaire) were consistent in their judgement as the consistency ratio (CR) of all risk factors was less than 0.1 (Supplementary Table S1). The mean consistency ratio among experts was 0.051 (range: 0.03–0.07; n = 9). According to the AHP results, poultry density was determined as the most important risk factor for AI outbreaks in Mali. Proximity to poultry markets was identified as the next most important risk factor, followed by in decreasing order, proximity to commercial poultry farms, proximity to water areas, and proximity to roads (Table 3).

### Avian influenza risk maps

**Figure 1.**
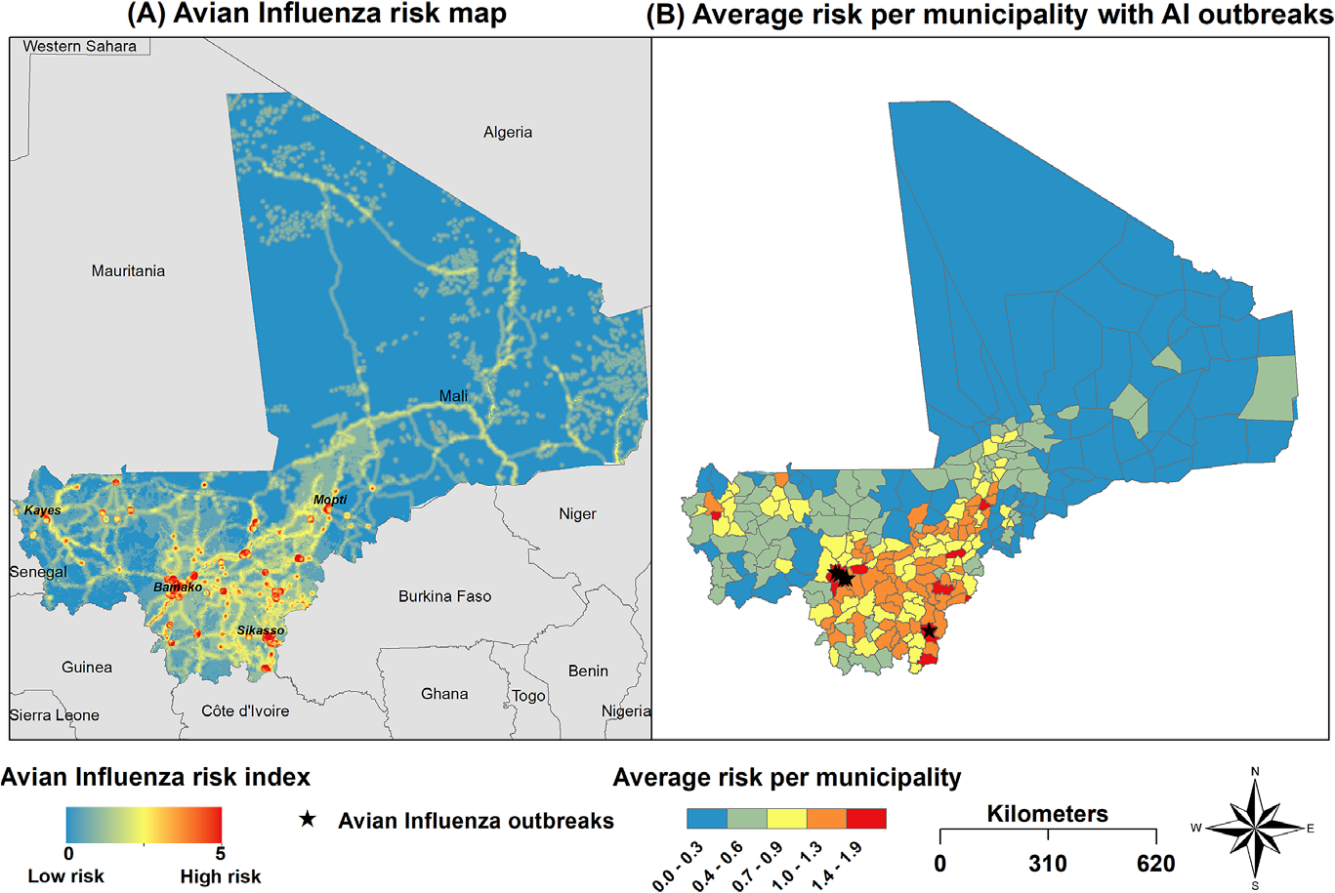
Map showing the risk of AIVs in domestic poultry in Mali on a continuous scale from low to high risk as defined by multi-criteria decision analysis. (A) Avian influenza risk map (B) Average risk per commune with the location of the seven avian influenza outbreaks in Mali.

In Figure 1(a), the risk of AI occurrence in domestic poultry in Mali was displayed on a continuous scale ranging from 0 (low risk, areas indicated with blue colour) to 5 (very high risk, areas indicated with red colour). Figure 1(b) represents the average risk of avian influenza outbreaks for municipalities. Areas with the highest risk for AI occurrence were located in the central (Mopti) and southeast regions (Bamako, Sikasso). Areas with moderate risk of AI occurrence included the southwest region (Kayes), neighbouring Senegal. In contrast, northern regions of Mali, including Tombouctou, Gao, and Kidal, were considered low-risk areas for AI occurrence.

### Uncertainty, sensitivity analysis and risk map validation

**Figure 2.**
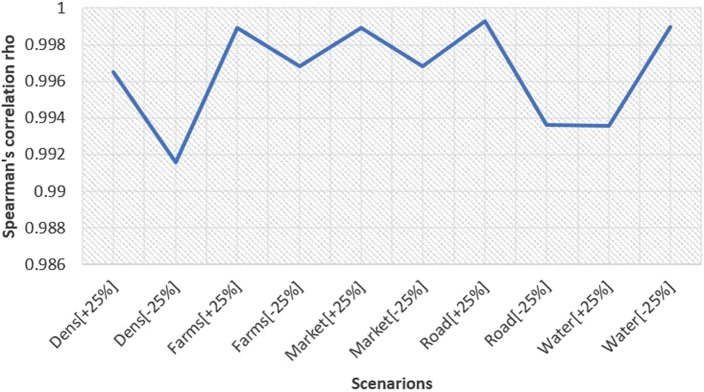
Spearman correlation coefficients (Rho) between raster cells of the avian influenza risk map and the ten scenarios in the sensitivity analysis. Dens, poultry density; Farms, poultry farms; Market, poultry markets; Road, proximity to roads; Water, proximity to water; [+25] and [-25] represent the increase and decrease in the relative weight of each risk factor, respectively.

**Figure 3.**
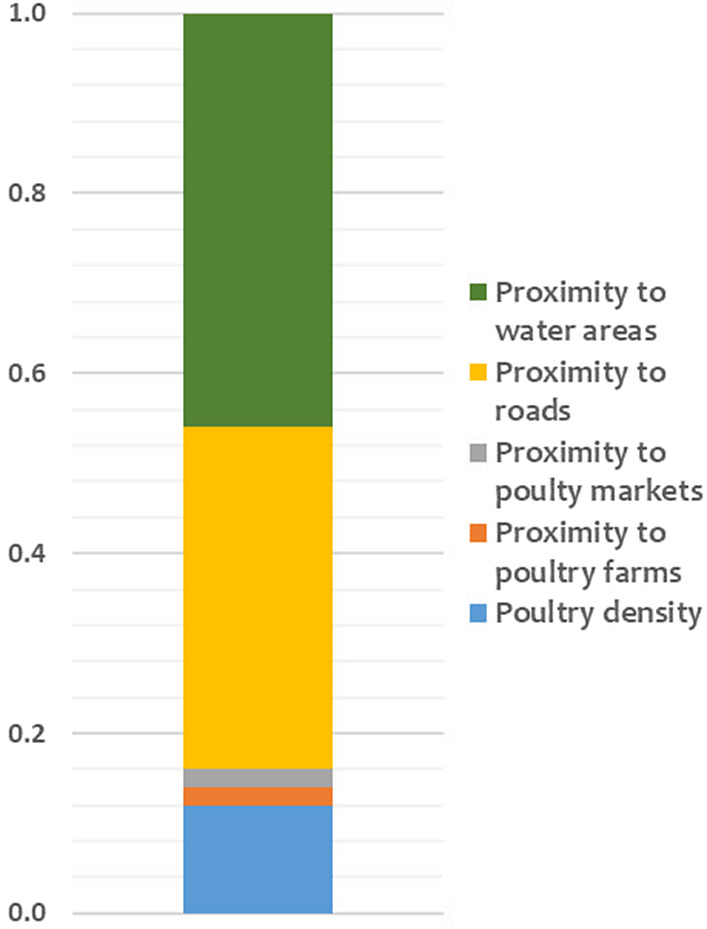
Contribution of risk factor weights to model output variance.

**Figure 4.**
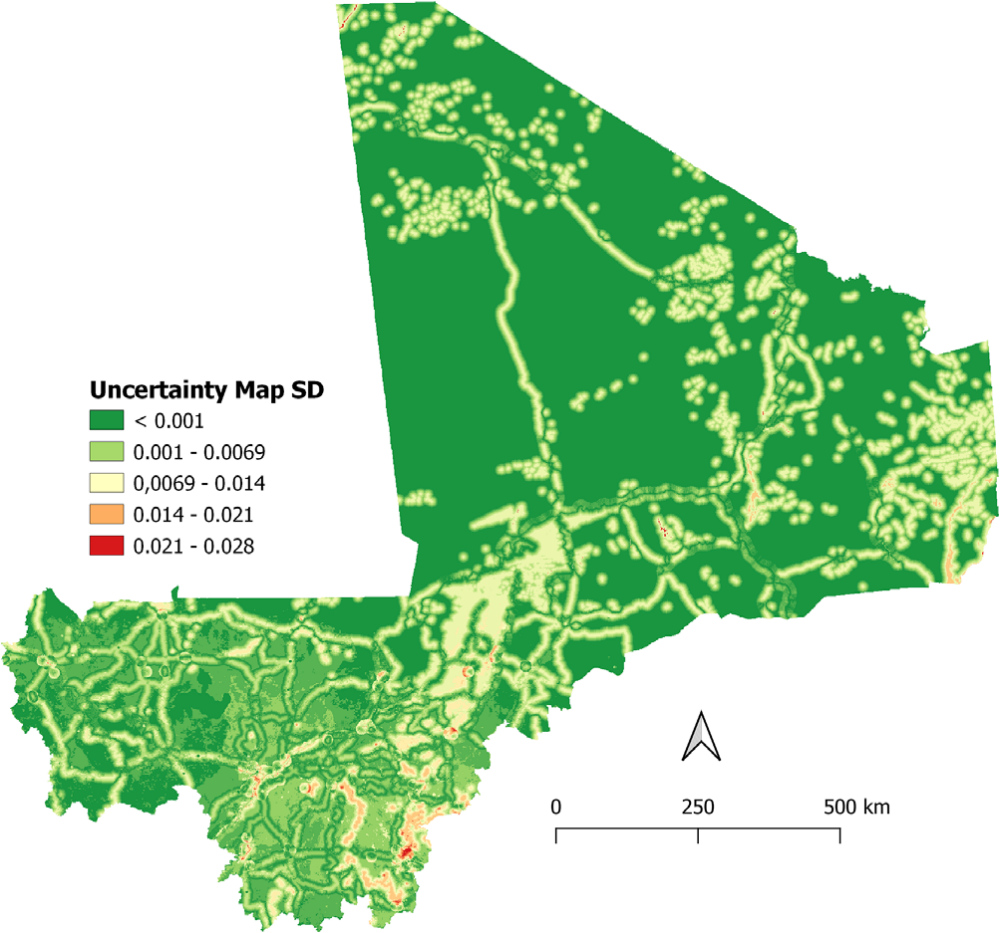
Uncertainty map (standard deviation of the risk maps for AI outbreaks in domestic poultry in Mali).

The results of the ten scenarios (Figure 2) performed in the sensitivity analysis were significantly correlated (Rho more than 0.99 and *p* < 0.001) with the final AI risk map. Therefore, the sensitivity analysis demonstrated that the model was robust, as changes in the relative weights of AI outbreak risk factors did not substantially modify the size and location of the areas determined as low, moderate, and high-risk areas for AIVs occurrence in Mali. The changes in the risk index of AIVs occurrence in domestic poultry in Mali were mainly explained by the weights of two risk factors (Figure 3). Indeed, proximity to water areas and proximity to roads were the most sensitive parameters, contributing to more than 60% of the model output variance. In contrast, the weights of proximity to poultry markets, proximity to commercial poultry farms, and poultry density were considered less sensitive. The maximum standard deviation of the uncertainty map (Figure 4) was less than 0.03, confirming that the predicted risk areas for AI outbreaks in Mali are robust, meaning that they remain stable when the weights of the risk factors are varied. The results highlighted a spatial heterogeneity of uncertainty, with higher uncertainty in areas of high risk for AI outbreaks.

A visual comparison of AI risk maps with the seven previous HPAI outbreak locations in Mali from EMPRES-i revealed that they all occurred in two municipalities deemed very high-risk areas for AI occurrence by our model (Figure 1(b)). Bamako and Mopti are also three areas identified as high-risk where influenza virus circulation has recently been reported [25].

## Discussion

This study presents the first risk maps for AIVs occurrence in domestic poultry in Mali. Areas with the highest risk for AI outbreaks were identified in Bamako District and Sikasso Region. The high density of domestic poultry and the high number of poultry markets and commercial poultry farms in those two regions could explain these results. Indeed, high poultry density and poultry markets have already been associated with an increased risk of AI occurrence [26–29]. In addition, commercial poultry farms are located mainly on the outskirts of major cities such as Bamako and Sikasso, with possible contact with backyard poultry and wild birds potentially infected by AIV. Interestingly, these high-risk areas for AIV occurrence overlap the locations of previous HPAI outbreaks reported in the country (EMPRES-i). Other regions of interest for AI occurrence in Mali include the central regions of Mali, particularly Mopti, which showed areas at high risk for AIV occurrence. A possible explanation for this might be the presence of the inner delta of the Niger River, which provides breeding and resting sites for millions of wild and migratory birds that are considered potential reservoirs for AIVs [11, 30].

The northern regions of Mali are extremely arid and characterized by low poultry density, a very limited number of poultry markets, and commercial poultry farms [13]. These factors may explain why the northern regions were determined by our model as at low risk of AI occurrence. However, the potential risk of AIV introduction through these areas via poultry movements should be considered since AI outbreaks are frequently reported from neighbouring Niger, which has not been taken into account by our model [8].

In this study, experts identified poultry density as the most important risk factor for AIV outbreaks in Mali. Several studies have reported an association between increasing poultry density and an increased risk of AIV outbreaks [27, 31]. Poultry density is associated with a higher contact rate between infected and susceptible birds and therefore a higher risk of spread. In addition, over 90% of domestic poultry in Mali are reared in traditional systems with limited biosecurity measures, which may explain why poultry density can represent a major risk factor for AIV occurrence. Notably, our results were not consistent with a previous study in Indonesia where poultry density was negatively correlated with AIV outbreaks [32]. However, this was probably because the highest poultry densities were found in areas with industrial farms with good management practices and strict biosecurity measures. The sensitivity analysis showed that proximity to water bodies and proximity to roads were the most sensitive factors, whose variation has the highest impact on the variability of the risk index. This finding implies that special attention should be paid to the accuracy and timeliness of these two factors when collecting primary spatial data in order not to significantly bias the risk assessment of AIV occurrence in Mali.

Several common limitations inherent to the GIS-MCDA approach have been described by different authors [17, 18, 20]. One of the main limitations of the present study resides in the selection of risk factors associated with AIVs occurrence. The risk factors integrated into the model were only those identified in the scientific literature to be associated with AI outbreaks and for which spatial data were available. Other risk factors such as poultry movements, seasons, husbandry practices, and wild bird distribution may play an important role in AIVs occurrence and were not included in our model due to a lack of available spatial data or unsuitability for GIS-MCDA [11, 33]. Particularly with regard to the risk factors associated with movement, network analysis, such as poultry trade flows, could be used as a complementary method to address the lack of data [34]. However, the model presented here is flexible and could be easily updated if data for other relevant factors for AIVs occurrence in Mali become available. Another limitation of the GIS-MCDA method is related to the quality of spatial data. In the present study, poultry density represents the most important risk factor. The raster layer available for poultry density in Mali was generated in 2014, 8 years before the study period, but it is unlikely that the density map has significantly evolved since then: the areas considered to have a high density of poultry in 2019 [35] were the same as those identified in the 2014 raster layer. More updated data would increase the quality of the resulting AI outbreak risk map in Mali.

A third limitation of the GIS-MCDA is the subjectivity that could be related to the weights assigned to risk factors. In the present study, the weights of risk factors were estimated using AIVs expert opinion elicitation. Due to the potential subjectivity that could be linked to the weights of risk factors, an extensive sensitivity analysis was conducted to evaluate the impact that variations in weights have on the model output. Our model is considered robust as the changes in weights did not greatly modify the resulting risk map. Although there is no specification about the number of experts to consult when eliciting health problems [36], only nine experts fully replied to the questionnaire. However, the judgements of all the experts were consistent (Supplementary Table S1). Unfortunately, a full validation of the model could not be performed. Although the visual overlay of the risk map with the areas where AIVs circulation has been reported is consistent, a quantitative validation using the ROC method could not be performed. In fact, quantitative validation of the model’s predictive capacity would require surveillance data from a larger number of areas identified as high risk, but also from areas identified as low risk, which are not currently available for Mali.. The validation of knowledge-driven models such as GIS-MCDA may be difficult, particularly in data-scarce settings. However, the MCDA approach has been validated in several studies [19, 20] and had even more predictive capacity than statistical models in certain situations [31]. In addition, data on AIV circulation from future studies and surveillance datasets could be easily integrated into the model for quantitative validation, even though collecting surveillance data in low-risk areas may remain challenging. Despite the drawbacks of the GIS-MCDA approach, its outputs could be particularly useful for veterinary services in implementing risk-based surveillance strategies and control programs in Mali or in other settings with very limited resources. In this perspective, GIS-MCDA can contribute to identifying risk areas for AI outbreaks, setting surveillance priorities, and efficiently allocating resources for the early detection and control of AI outbreaks. However, when using the GIS-MCDA approach to guide risk-based surveillance, careful consideration should be given to the sensitivity and robustness of the model, and AI outbreak prevention and control measures should be designed according to local conditions, such as husbandry practices, spatial distribution of local poultry farms, live bird trade, and organization of poultry markets. In addition, as more quantitative data on AI risk factors becomes available, the risk map and the relative weights of each risk factor should be re-evaluated to improve the accuracy of risk modelling. In conclusion, this study provides a cost-effective tool to guide future surveillance activities in Mali and also presents a methodological approach that could be implemented in other regions similar to Mali, particularly in Africa, to prevent and mitigate the occurrence of AIVs.

## Supporting information

Sanogo et al. supplementary material 1 Sanogo et al. supplementary material

Sanogo et al. supplementary material 2 Sanogo et al. supplementary material

## Data Availability

Data are available upon request from the corresponding authors.

## Supplementary material

The supplementary material for this article can be found at http://doi.org/10.1017/S0950268824001390.
