# Supplementary material for: Identification of risk areas for avian influenza outbreaks in domestic poultry in Mali using the GIS-MCDA approach
Source: Epidemiol Infect. 2024 Dec 2;152:e152. doi: 10.1017/S0950268824001390 (PMC11626446; doi:10.1017/S0950268824001390)
Supplement: Sanogo et al. supplementary material 1 — Sanogo et al. supplementary material [file S0950268824001390sup001.docx]

*Epidemiology and Infection*

Identification of risk areas for Avian Influenza outbreaks in poultry in Mali using the GIS-MCDA approach

# Supplementary Materials

Table S1: Relative weights of risk factors assigned by experts

| Criteria | Poultry density | Poultry farms | Poultry Market | Proximity to roads | Proximity to water | Total | CR Value* |
| --- | --- | --- | --- | --- | --- | --- | --- |
| Expert 1 | 0.34 | 0.34 | 0.09 | 0.10 | 0.12 | 1 | 0.07 |
| Expert 2 | 0.29 | 0.15 | 0.44 | 0.06 | 0.06 | 1 | 0.05 |
| Expert 3 | 0.42 | 0.06 | 0.25 | 0.13 | 0.13 | 1 | 0.05 |
| Expert 4 | 0.26 | 0.33 | 0.22 | 0.07 | 0.12 | 1 | 0.07 |
| Expert 5 | 0.26 | 0.33 | 0.22 | 0.12 | 0.07 | 1 | 0.07 |
| Expert 6 | 0.28 | 0.18 | 0.35 | 0.12 | 0.07 | 1 | 0.05 |
| Expert 7 | 0.28 | 0.28 | 0.28 | 0.12 | 0.04 | 1 | 0.04 |
| Expert 8 | 0.24 | 0.24 | 0.3 | 0.12 | 0.1 | 1 | 0.03 |
| Expert 9 | 0.45 | 0.16 | 0.16 | 0.17 | 0.06 | 1 | 0.03 |
| Mean % | **31.33** | **23.07** | **25.62** | **11.33** | **8.65** |  | **0.05** |

*Consistency Ratio

**
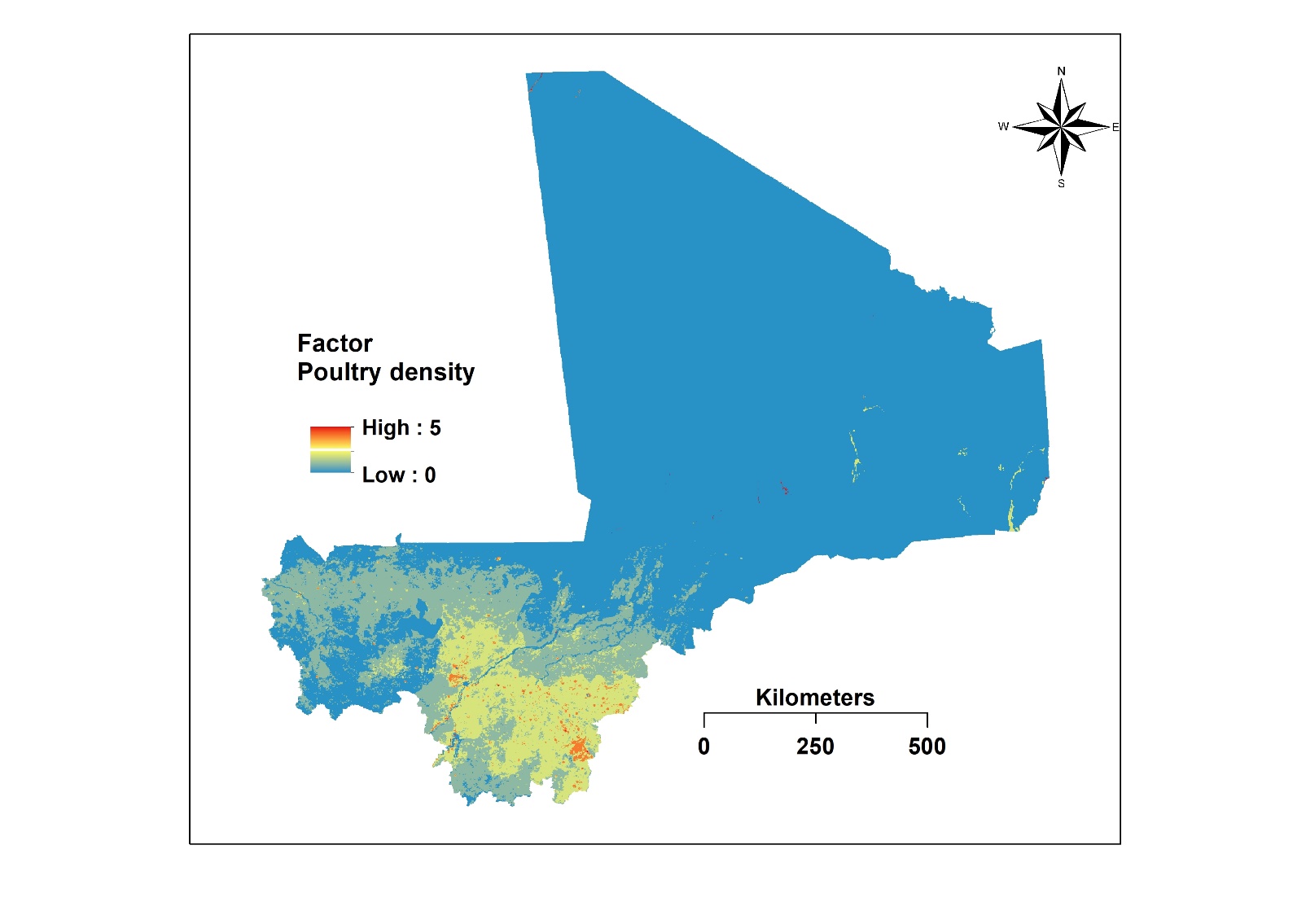
**

Figure S1: Map of poultry density after standardization.


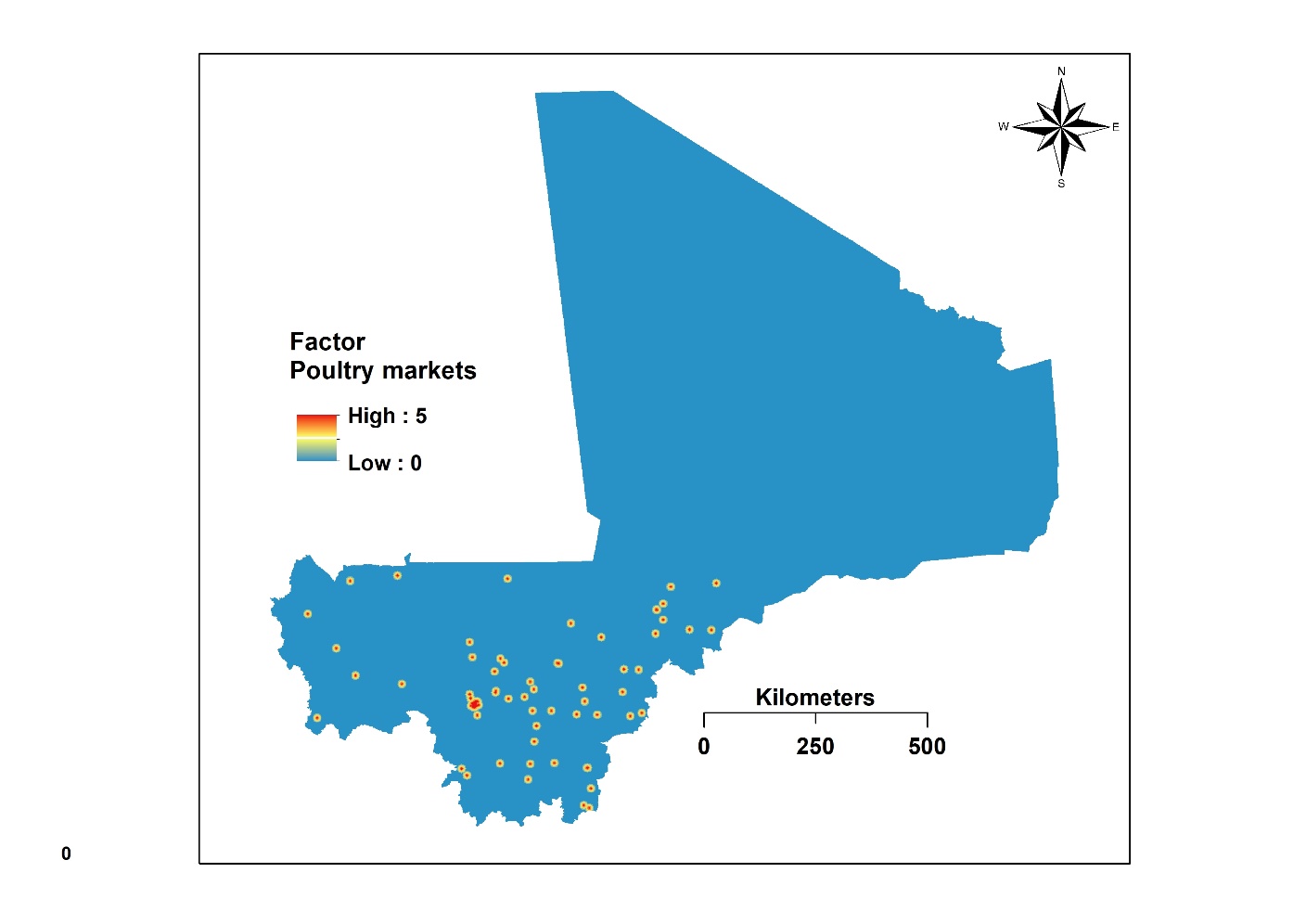


Figure S2: Map of distance to livestock market after standardization.


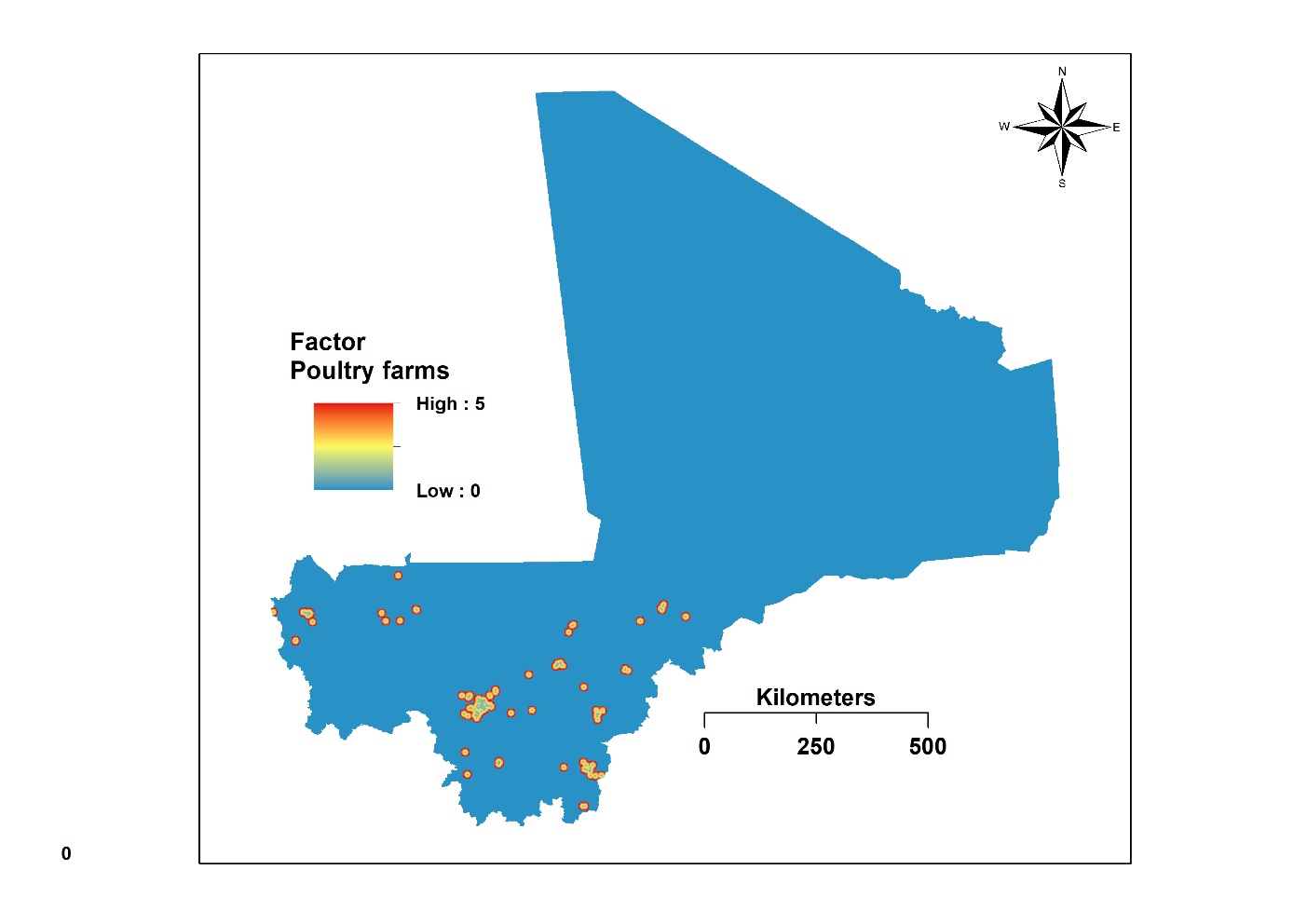


Figure S3: Map of distance to poultry farms after standardization.


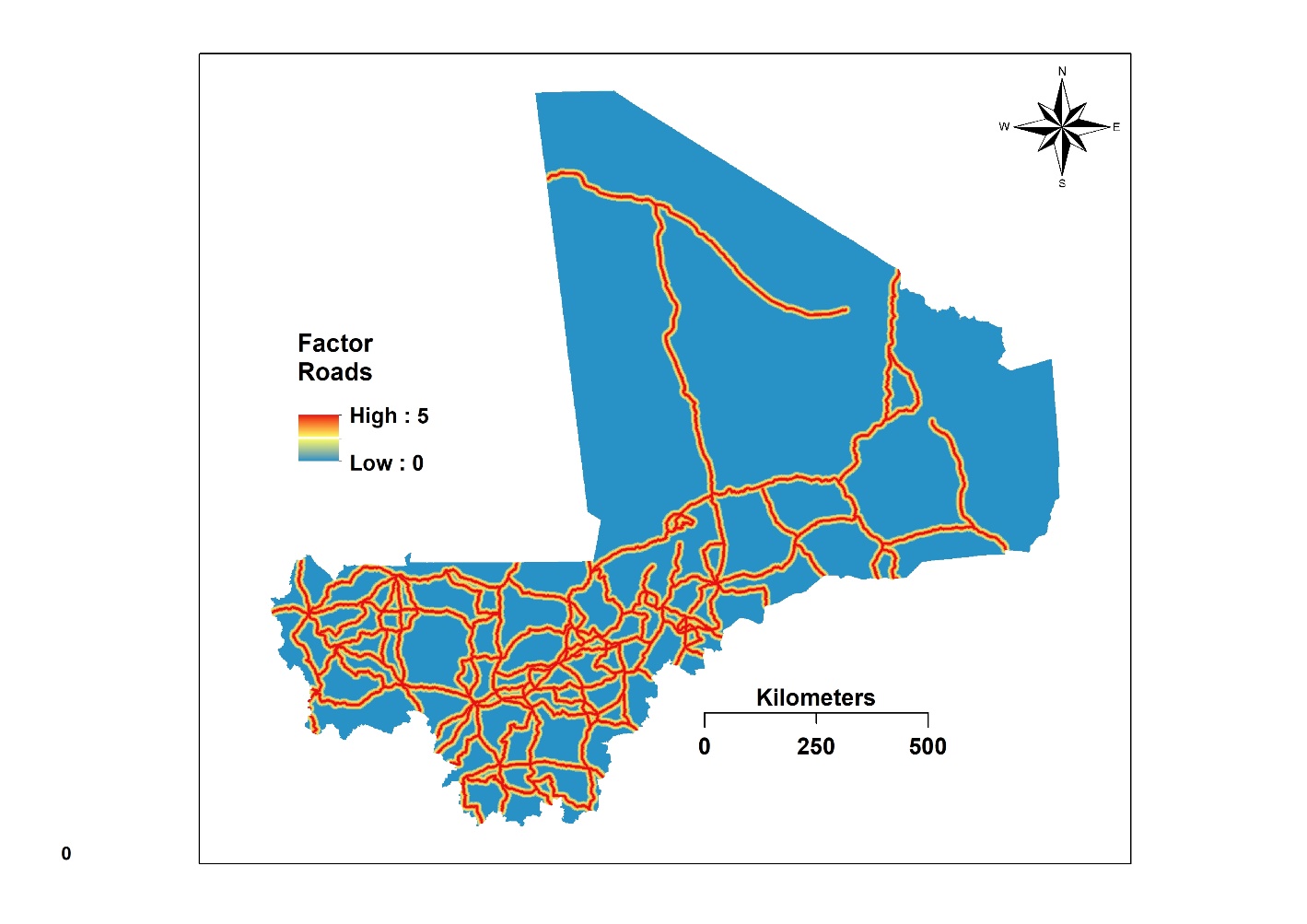


Figure S4: Map of distance to roads after standardization.


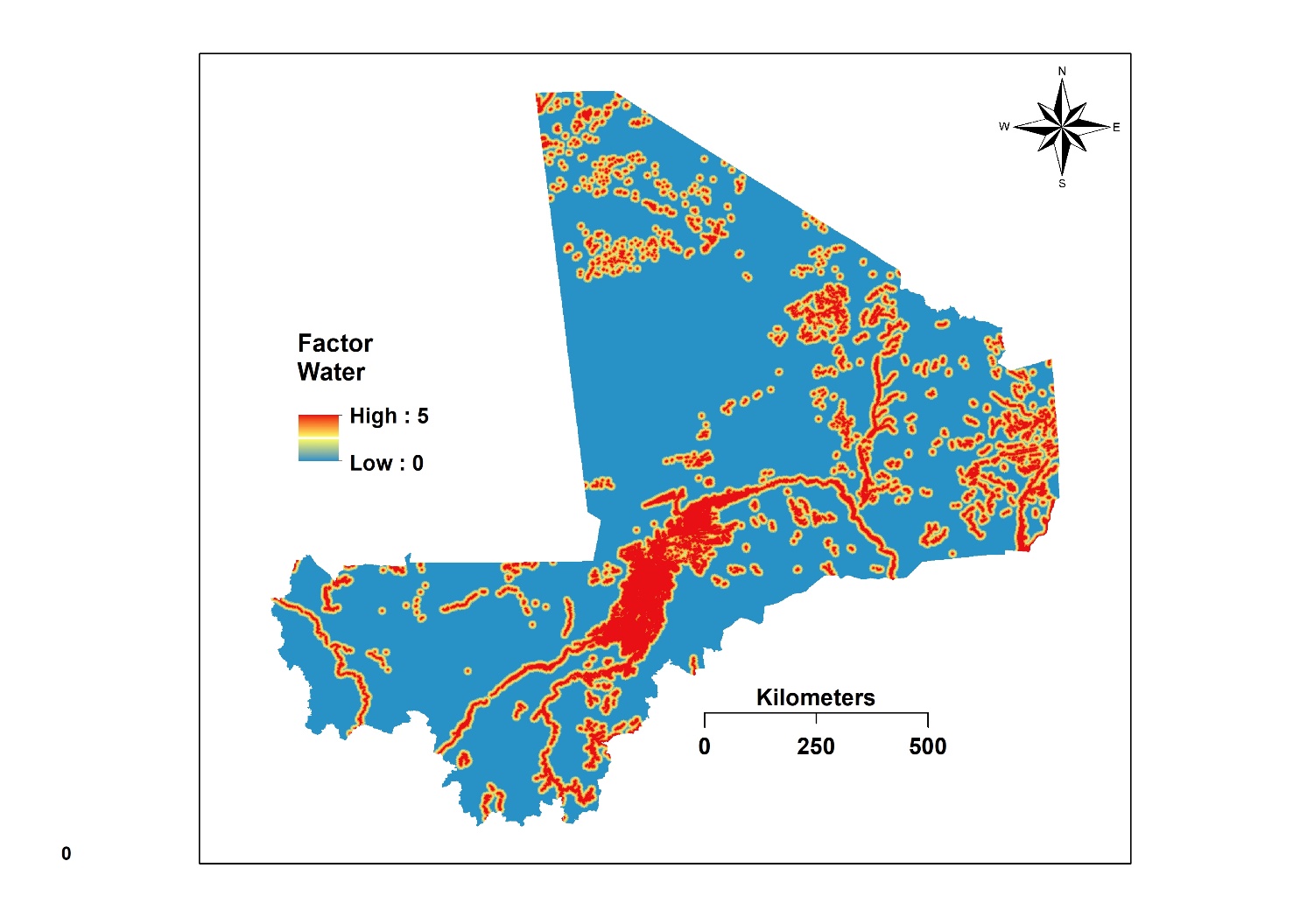


Figure S5: Map of distance to water areas after standardization.
