# Supplementary material for: Identification of risk areas for avian influenza outbreaks in domestic poultry in Mali using the GIS-MCDA approach
Source: Epidemiol Infect. 2024 Dec 2;152:e152. doi: 10.1017/S0950268824001390 (PMC11626446; doi:10.1017/S0950268824001390)
Supplement: Sanogo et al. supplementary material 2 — Sanogo et al. supplementary material [file S0950268824001390sup002.pdf]

Risk analysis of the occurrence of avian influenza virus (AI) in Mali with the GIS-based Multicriteria decision analysis (GIS-MCDA)

We intend to evaluate the occurence of avian influenza viruses in Mali by using the GIS-MCDA method. The aim of this form is to determine the main risk factors contributing to the occurence of AI in Mali as well as their relative importance and relations with the risk of AI.

* Indicates required question

1. Name and Surname - Functions *

Risk factors for the occurence of AI

According to certain studies realized in Africa, risk factors listed below contribute to the occurence of AI.
In your opinion are these risk factors important for avian influenza in Mali?

2. Poultry density *

Mark only one oval.

- Yes
- No

3. Population density *

Mark only one oval.

- Yes
- No

4. Proximity to poultry markets *

Mark only one oval.

- Yes
- No

5. Proximity to main roads *

Mark only one oval.

- Yes
- No

6. Proximity to open waters *

Mark only one oval.

- Yes
- No

7. Other risk factors

Determining the importance of risk Factors

In this section you should estimate the relative importance of each risk factor compared to other risk factors in relation to its contribution to the risk of occurrence of AI in Mali.

8. 1. Poultry density is a risk factor *

5 = Much more important than 3 = Moderately more important than 1= Equally important as 1/3 = Moderately less important than 1/5 = Much less important than

Mark only one oval per row.

	Much more important than (5)	Moderately more important than (3)	Equally important as (1)	Moderately less important than (1/3)	Much less important than (1/5)
**Population density**	◯	◯	◯	◯	◯
**Proximity to poultry markets**	◯	◯	◯	◯	◯
**Proximity to main roads**	◯	◯	◯	◯	◯
**Proximity to opens waters**	◯	◯	◯	◯	◯

9. 2. Population density is a risk factor *

5 = Much more important than 3 = Moderately more important than 1= Equally important as 1/3 = Moderately less important than 1/5 = Much less important than

Mark only one oval per row.

	Much more important than (5)	Moderately more important than (3)	Equally important as (1)	Moderately less important than (1/3)	Much less important than (1/5)
**Poultry density**					
**Proximity to poultry markets**					
**Proximity to main roads**					
**Proximity to opens waters**					

10. 3. Proximity to poultry markets is a risk factor *

5 = Much more important than 3 = Moderately more important than 1= Equally important as 1/3 = Moderately less important than 1/5 = Much less important than

Mark only one oval per row.

	Much more important than (5)	Moderately more important than (3)	Equally important as (1)	Moderately less important than (1/3)	Much less important than (1/5)
**Poultry density**					
**Population density**					
**Proximity to main roads**					
**Proximity to opens waters**					

11. 4. Proximity to main roads is a risk factor *

5 = Much more important than 3 = Moderately more important than 1= Equally important as 1/3 = Moderately less important than 1/5 = Much less important than

Mark only one oval per row.

	Much more important than (5)	Moderately more important than (3)	Equally important as (1)	Moderately less important than (1/3)	Much less important than (1/5)
**Poultry density**	◯	◯	◯	◯	◯
**Population density**	◯	◯	◯	◯	◯
**Proximity to poultry markets**	◯	◯	◯	◯	◯
**Proximity to opens waters**	◯	◯	◯	◯	◯

12. 5. Proximity to opens waters *

5 = Much more important than 3 = Moderately more important than 1= Equally important as 1/3 = Moderately less important than 1/5 = Much less important than

Mark only one oval per row.

	Much more important than (5)	Moderately more important than (3)	Equally important as (1)	Moderately less important than (1/3)	Much less important than (1/5)
**Poultry density**	◯	◯	◯	◯	◯
**Population density**	◯	◯	◯	◯	◯
**Proximity to poultry markets**	◯	◯	◯	◯	◯
**Proximity to main roads is a risk factor**	◯	◯	◯	◯	◯

Relations between risk factors and the occurrence of Avian Influenza

Illustration by examples of the possible relationships between risk factors and the occurrence of AI.

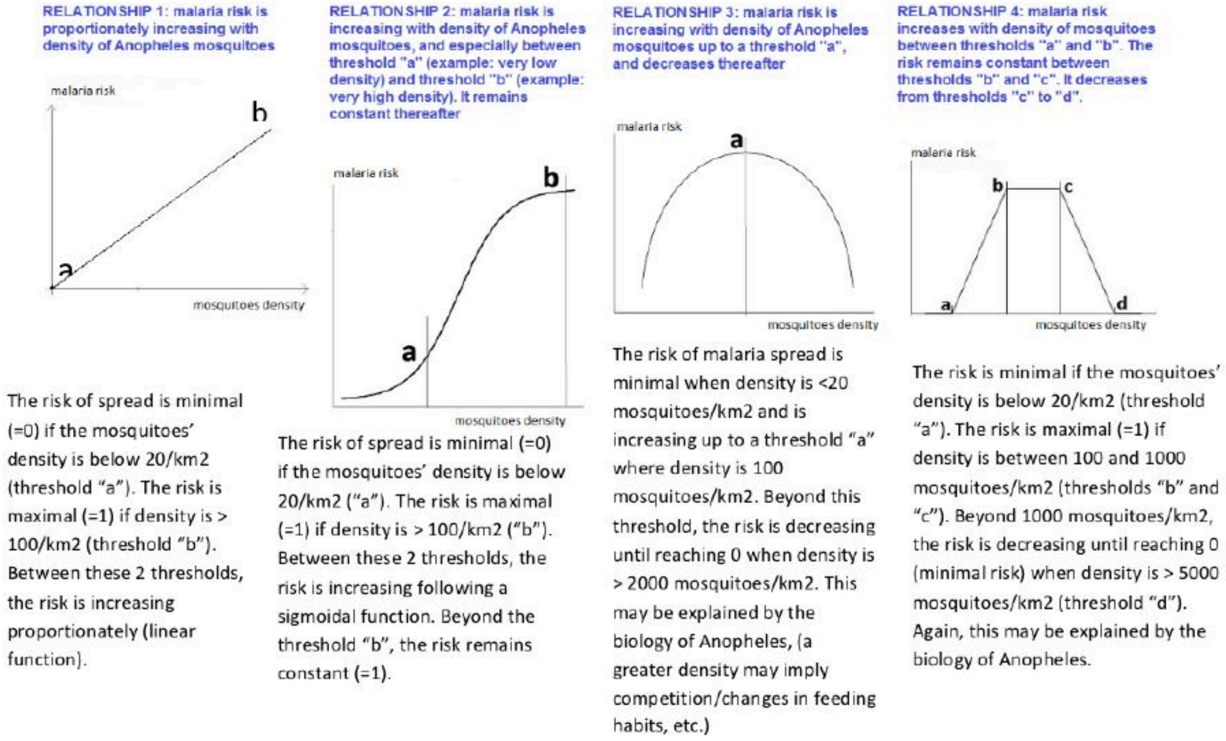

13. 1. Poultry density has a relationship... *

Mark only one oval.

- Relationship 1
- Relationship 2
- Relationship 3
- Relationship 4

14. What could be the values of a and b (if "relationship 4 " the values of c and d)?

15. 2. Population density has a relationship... *

Mark only one oval.

- Relationship 1
- Relationship 2
- Relationship 3
- Relationship 4

16. What could be the values of a and b (if "relationship 4 " the values of c and d)?

____

17. 3. Proximity to poultry markets has a relationship... *

Mark only one oval.

- Relationship 1
- Relationship 2
- Relationship 3
- Relationship 4

18. What could be the values of a and b (if "relationship 4 " the values of c and d)?

____

19. 4.Proximity to main roads has a relationship... *

Mark only one oval.

- Relationship 1
- Relationship 2
- Relationship 3
- Relationship 4

20. What could be the values of a and b (if "relationship 4 " the values of c and d)?

____

21. What is the risk in terms of proportion (%) of the risk linked to the proximity of the roads that could be attributed to primary and secondary roads?

____

22. 5. Proximity to open waters has a relationship... *

Mark only one oval.

- Relationship 1
- Relationship 2
- Relationship 3
- Relationship 4

23. What could be the values of a and b (if "relationship 4 " the values of c and d)?

____

24. Do you have any comments about the study?

____
____
____
____
____

This content is neither created nor endorsed by Google.

Google Forms
